# *Ginkgo biloba* Extract Modulates the Retroperitoneal Fat Depot Proteome and Reduces Oxidative Stress in Diet-Induced Obese Rats

**DOI:** 10.3389/fphar.2019.00686

**Published:** 2019-06-14

**Authors:** Bruna K.S. Hirata, Amanda P. Pedroso, Meira M.F. Machado, Nelson I.P. Neto, Bruna O. Perestrelo, Roberta D.C.C. de Sá, Maria Isabel C. Alonso-Vale, Fernando N. Nogueira, Lila M. Oyama, Eliane B. Ribeiro, Alexandre K. Tashima, Monica M. Telles

**Affiliations:** ^1^Department of Biological Sciences, Universidade Federal de São Paulo–UNIFESP, Diadema, Brazil; ^2^Department of Physiology, Universidade Federal de São Paulo–UNIFESP, São Paulo, Brazil; ^3^Department of Biomaterials and Oral Biology, School of Dentistry, Universidade de São Paulo–USP, São Paulo, Brazil; ^4^Department of Biochemistry, Universidade Federal de São Paulo–UNIFESP, São Paulo, Brazil

**Keywords:** *Ginkgo biloba*, proteome, retroperitoneal adipose tissue, oxidative stress, obesity, rats

## Abstract

The rapid increase in the number of individuals with obesity, over the past four decades, is triggered by a number of complex interactions among factors. Despite the plethora of treatments available, side effects are commonly observed and, in this context, herbal medicines have been employed as an alternative form of therapy. *Ginkgo biloba* extract (GbE) has been described as a promising new pharmacological approach to treat obesity. In order to better comprehend the mechanisms involved with this potential effect, the present study evaluated the effects of GbE treatment on diet-induced obese rats, focusing on the proteome and the oxidative stress defense system of visceral adipose tissue. After 14 days treatment, GbE significantly modulated 25 proteins. Retroperitoneal adipose tissue of treated animals exhibited higher amounts of proteins associated with adipogenesis (decorin), carbon metabolism and mitochondrial function (citrate synthase), and a concomitant reduction in adipocyte hypertrophy. In parallel, GbE down-regulated proteins involved in oxidative stress (peroxiredoxin) and the inflammatory response (complement C3, mast cell protease 1, and Ig gamma-2B chain C region). Moreover, also related to oxidative stress defense, GbE stimulated catalase activity, reduced malondialdehyde levels (lipid peroxidation indicator), and increased lactoylglutathione lyase levels. It was concluded that GbE acts as an antioxidant agent, and improved the proteome profile and oxidative stress response in the adipose tissue of diet-induced obese rats.

## Introduction

According to World Health Organization, obesity is a chronic disease that has dramatically increased since 1975, and has become a primary health concern (NCD-RisC, 2017; World Health Organization, 2018). It is known that high-fat diet intake leads to deleterious metabolic effects, including abnormal adipocyte hypertrophy and subsequent hypoxia, as well as oxidative stress and low-grade chronic inflammation, especially in the visceral white adipose tissue depots (Manna and Jain, 2015; Francisqueti et al., 2017). Growing evidence suggests that oxidative stress and chronic inflammation, resulting from obesity, can promote the development of complications such as insulin resistance, type 2 diabetes, cardiovascular complications, hepatic dysfunction, and carcinogenesis (Rupérez et al., 2014; Manna and Jain, 2015).

Oxidative stress is defined as an imbalance between pro-oxidant and anti-oxidant systems, resulting in the overproduction of free radicals and reactive oxygen species (ROS) (Rani et al., 2016). Despite the fact that ROS play an important role in the regulation of intracellular signaling processes, by serving as secondary messengers (Yoshikawa and Naito, 2002; Burton and Jauniaux, 2011), excessive ROS generation irreversibly modifies lipids, proteins, and DNA (Burton and Jauniaux, 2011; Rani et al., 2016).

A number of studies have investigated the efficacy of alternative therapies in the management of metabolic diseases (de Freitas Junior and de Almeida Jr., 2017; Perumpail et al., 2018). In fact, *Ginkgo biloba* extract (GbE) is one of the most commonly used therapies worldwide and has been reported to be a promising new pharmacological approach for treating obesity. Recent data from our laboratory demonstrated the potential use of GbE for treating obesity, as well as obesity-induced insulin resistance. More specifically, GbE reduced body weight gain and the food/energy intake, and also improved insulin sensitivity in the visceral adipose tissue and gastrocnemius muscle of diet-induced obese (DIO) male rats (Banin et al., 2014; Hirata et al., 2015). Additionally, GbE stimulated the hypothalamic serotonergic system and attenuated obesity in ovariectomized rats (Banin et al., 2017).

It is thought that GbE has a potent antioxidant effect, due to its ability to scavenge free radicals and ROS, consequently inhibiting the formation and accumulation of these dangerous molecules (Marcocci et al., 1994; Mahadevan and Park, 2008). Standardized GbE contains 24% flavone glycosides and 6% terpenoids (Drieu, 1986), both of which have demonstrated antioxidant activity, and have been shown to increase antioxidant enzyme activity and reduce lipid peroxidation, under different experimental conditions (Rojas et al., 2011; Boghdady, 2013; Priyanka et al., 2014; Yallapragada and Velaga, 2015). In order to better understand the molecular mechanisms underlying these previously observed effects, the present study investigated the effects of GbE on the retroperitoneal fat depot proteome and oxidative stress defense system in DIO rats.

## Methods

### Ethical Approval

The study was designed and carried out in strict accordance with the recommendations set forth by the Guide for the Care and Use of Laboratory Animals (2011). The protocol was approved by the Universidade Federal de São Paulo Animal Research Ethics Committee (process number: 8700110814).

### Phytotherapy Treatment

**Figure 1:**
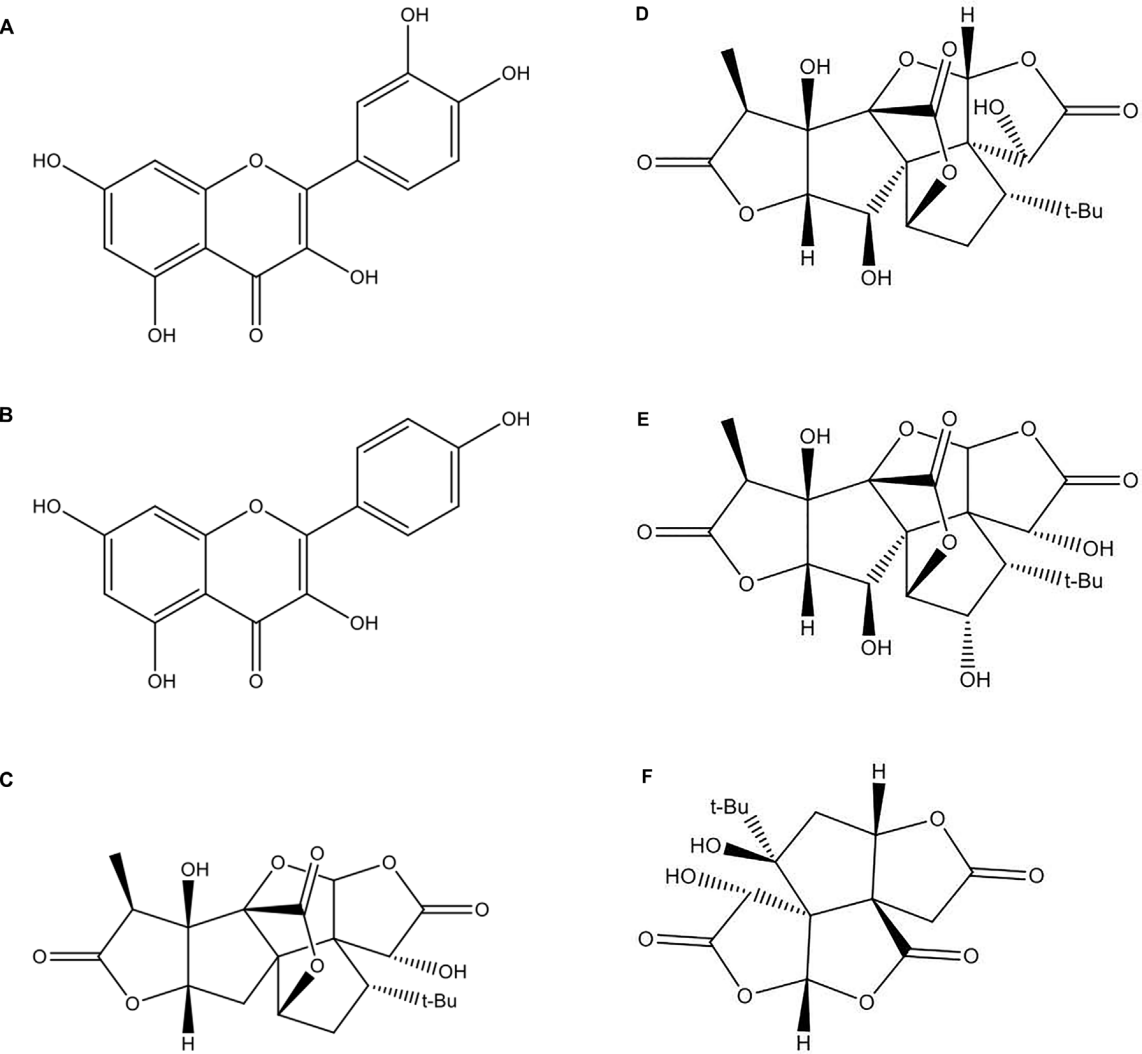
The chemical structures of the *Ginkgo biloba* extract (GbE) based on the high-performance liquid chromatography (HPLC) profile of the extract performed by the manufacturer: **(A)** quercetin; **(B)** kaempferol; **(C)** gingkolide A; **(D)** ginkgolide B; **(E)** ginkgolide C; **(F)** (-) bilobalide.

The standardized GbE was obtained from Huacheng Biotech Inc. (China) and contained 25.21% flavone glycosides and 6.62% terpene lactones (3.09% ginkgolides A, B, C, and 2.73% bilobalides), based on the high-performance liquid chromatography (HPLC) profile of the extract performed by the manufacturer. The chemical structures of the pharmacoactive compounds are depicted in **Figure 1**.

Two-month-old male Wistar rats, obtained from the Multidisciplinary Center for Biological Investigation in Laboratory Animals Science (CEMIB—Campinas, Brazil), were housed at four rats per cage, provided with food and water *ad libitum*, and maintained in a temperature-controlled room (23°C ± 1°C), under a 12:12-h light/dark cycle (lights on at 6 am).

As previously described (Banin et al., 2014; Hirata et al., 2015), the high-fat diet (HFD) was prepared by mixing 40% (w/w) standard chow with 28% (w/w) melted lard, 20% (w/w) casein powder, 10% (w/w) sucrose, 2% (w/w) soybean oil, and 0.02% (w/w) butylated hydroxytoluene (5.0 kcal/g). All of the animals were fed the HFD for 2 months and were then divided into two groups. One group was administered 500 mg/kg of GbE (HFD+GbE) (Oliveira et al., 2009; Banin et al., 2014; Hirata et al., 2015; Banin et al., 2017), and the other group received 0.9% saline (HFD).

Following the experimental period, all of the animals were fasted for 8 h, anaesthetized (barbiturate 80 mg/kg), and euthanized by decapitation.

### Antioxidant Enzyme Activities

Retroperitoneal adipose tissue was weighed and homogenized in phosphate buffer (100 mM Tris, pH 7.5; 10 mM EDTA; 10 mM Na_3_VO_4_; 100 mM NaF; 10 mM Na_4_P_2_O_7_; 2 mM PMSF; and 0.1 mg/mL aprotinin). Glutathione peroxidase (GPx) activity was measured using a commercially available Ransel assay (product #RS504, Randox Laboratories, UK), and performed accordingly to manufacturer’s instructions. Catalase (CAT) activity was determined spectrophotometrically (Beckman DU640), by calculating the rate of H_2_O_2_ decay at 240 nm, as described previously (Aebi, 1984). The protein concentrations were determined using the Bradford method (Bradford, 1976).

### Determination of Free Malondialdehyde

Lipid peroxidation was assessed using the method of Karatas et al. (2002). Briefly, retroperitoneal adipose tissue was homogenized in 0.4 M HClO_4_, centrifuged, and subjected to reversed-phase HPLC analysis, using a C18 column (15 cm × 4.6 mm × 3 µm). The mobile phase was composed of 50 mM KH_2_PO_4_-methanol (65:35) buffer and the eluate was monitored at 254 nm. Levels of MDA were calculated by extrapolating the observed values to an MDA standard curve, and data were expressed as MDA/mg protein.

### Evaluation of Adipocyte Volume

Retroperitoneal adipose tissue was immediately collected following decapitation. The tissue was diced and mixed with 4 ml of DMEM supplemented with 20 mM HEPES, 5 mM glucose, 1% bovine serum albumin (BSA), and 1 mg/ml collagenase type II, pH 7.4, and incubated on an orbital shaker for 40 min at 37°C. Isolated adipocytes were filtered and washed three times with DMEM solution. The harvested adipocytes were photographed using an optic microscope (100× magnification) coupled to a microscope camera (Axiocam ERc5s; Zeiss^®^, Oberkochen, Germany). The mean adipocyte diameter of 50 cells was measured using the AxioVision LE64 software, and adipocyte volume was calculated using the following formula: 4/3×π×r^3^ (Randle, 1972; Bolsoni-Lopes and Alonso-Vale, 2015; de Sá et al., 2016).

### Proteomic Analyses

Each fat depot specimen from each animal was prepared and analyzed independently (i.e., not pooled). Retroperitoneal fat pads were removed and homogenized in 1 ml of lysis buffer, containing: 8 M urea, 75 mM NaCl, 1 M Tris, and complete Mini Protease Inhibitor Cocktail Tablets (Roche Diagnostics, USA). Samples were then centrifuged at 19,000 × *g* for 30 min at 4°C. Total protein concentration was determined using the Lowry method (Lowry et al., 1951). As previously described (Pedroso et al., 2017), aliquots corresponding to 2,000 µg of total protein were transferred to Amicon Ultra-4 Centrifugal 3,000 NMWL filter devices (Merck Millipore), for buffer exchange into 50 mM NH_4_HCO_3_. Next, the protein concentration was measured again, and 200 µg of total protein was added to 25 µl of 0.2% RapiGest SF solution (Waters, USA) and incubated for 15 min at 80°C. Samples were then reduced with 5 mM DTT for 30 min at 60°C, and alkylated with 10 mM iodoacetamide, in the dark for 30 min at room temperature. Proteins were digested with trypsin Gold (Promega, USA) at a protease/protein ratio of 1:100 (w/w), overnight at 37°C. Digestions were terminated by adding 10 µl of 5% trifluoroacetic acid (TFA) for 90 min at 37°C. Samples were centrifuged at 19,000 × *g* for 10 min at 4°C, and the supernatants were collected. The final protein concentrations were typically around 2 µg/µl.

### Mass Spectrometry (Data Independent Acquisition Mode)

Digested samples (six biological replicates per group, three technical replicates) were analyzed using a Synapt G2 HDMS Q-TOF mass spectrometer (Waters) coupled to a nanoAcquity UPLC chromatographic system, as previously described by Pedroso et al. (2017). Samples were injected onto a nanoAquity C18 Symmetry trap column (180 µm × 20 mm, Waters) and transferred with an elution gradient to a nanoAcquity C18 BEH analytical column (75 µm × 250 mm, 1.7 mm, Waters). Buffers A (0.1% formic acid in water) and B (0.1% formic acid in acetonitrile) were used to generate a 7–35% buffer B elution gradient run over 92 min at a flow rate of 275 nl/min. Data were acquired in the HDMSE mode, alternating between low (4 eV) and high (ramped from 19 to 45 eV) collision energies, which provided an accurate measurement of mass for both intact peptides and fragments. For external calibration, Glu-fibrinopeptide B (Waters) was infused into a NanoLockSpray apparatus and scanned every 30 s. The ProteinLynx Global Server version 3.0.1 software (Waters) was used for mass spectrometry data processing and results were searched against *Rattus norvegicus* sequences in the UniProtKB/Swiss-Prot database (www.uniprot.org). Database search parameters included: cysteine carbamidomethylation as a fixed modification, methionine oxidation, as variable modifications; up to two missed cleavage sites were allowed for trypsin digestion and automatic fragment and peptide mass tolerance. Protein identification criteria included a minimum of one fragment ion per peptide, five fragment ions per protein, two peptides per protein and the false discovery identification rate was set to 1%, estimated by a simultaneous search against a randomized database. Label-free quantitative assessments based on peptide intensities were performed. The top 3 matched peptides (integrated intensities of the three most intense peptides of each identified protein) were used for relative quantitation. The analysis only included proteins identified in at least three biological replicates and two technical replicates. Moreover, proteins absent in one group and identified in at least 4 out of 12 replicates in the other group were considered to be exclusive proteins. Normalization was performed in each sample according to the sum of protein intensities.

The integration of protein–protein interactions were investigated using the String search tool (STRING v10.5) (Szklarczyk et al., 2015).

### Statistical Analysis

All results are presented as the mean ± standard error of the mean (SEM). Differences between the HFD+GbE group and the obese HFD group were determined using an unpaired Student’s t-test. Statistical analyses were performed using the SPSS^®^ software version 20.0 (IBM, Chicago, USA). Results were considered to be statistically significant when p < 0.05.

### Availability of Data and Material

All data generated or analyzed during the course of this study are included in this published article as supplementary information files, and are available in the repository.

## Results

### GbE Treatment Augmented Catalase Activity and Reduced MDA Levels in Retroperitoneal Adipose Tissue

**Figure 2:**
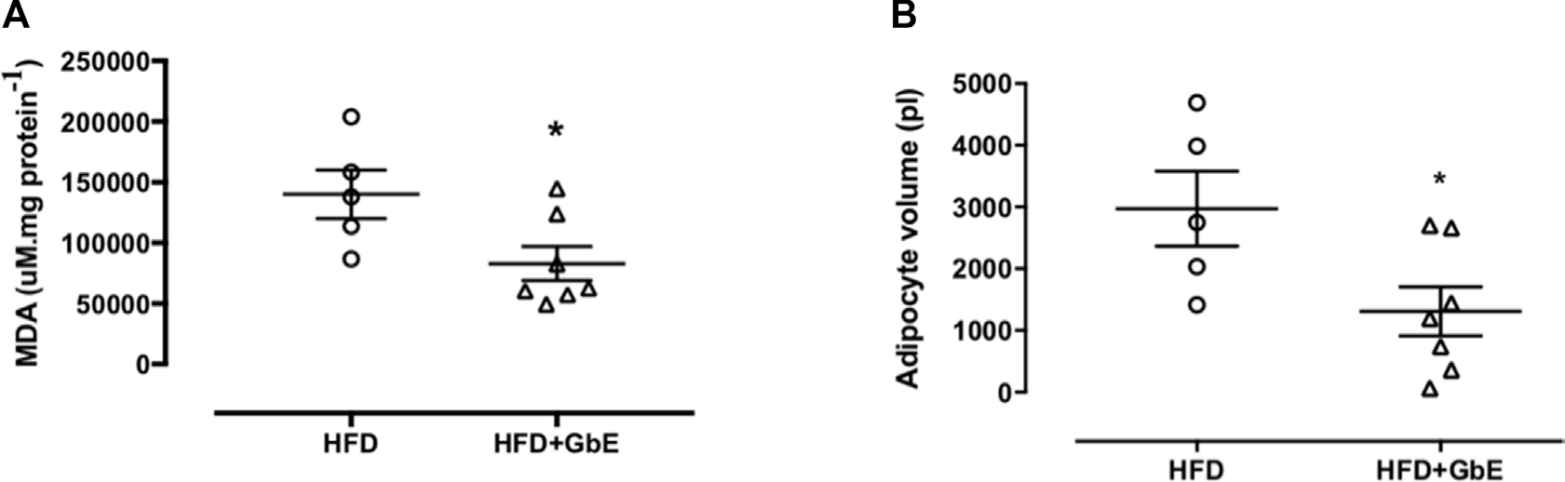
**(A)** Levels of malondialdehyde (MDA) measured in retroperitoneal adipose tissue of the HFD and HFD+GbE groups (µM/mg protein); **(B)** retroperitoneal adipocyte volume of the HFD and HFD+GbE groups (ρL). Values are expressed as the mean ± SEM (n = 5–7).

As shown in **Table 1**, the HFD+GbE group displayed a significant 30% increase in catalase activity, when compared to animals fed a HFD. However, there was no observable difference in GPx activity. In addition to the augmented catalase activity, the levels of MDA, a secondary product of lipid peroxidation, were decreased by 40% in the HFD+GbE group, when compared to the HFD group (**Figure 2A**). Together these results demonstrate an upregulation in antioxidant activity and further validated the antioxidant potential of GbE.

**Table 1:** Antioxidant enzyme activities.

Enzymes (Units. Mg protein^-1^)	HFD	HFD+GbE
GPx	0.084 ± 0.02	0.080 ± 0.01
CAT	14.3 ± 1.2	18.6 ± 1.0*

Data are expressed as the mean ± SEM (n = 6–7). *p < 0.05 was considered significantly different. GPx, glutathione peroxidase; CAT, catalase.

### GbE Treatment Reduced Adipocyte Volume and Modulated the Retroperitoneal Fat Depot Proteome

GbE treatment reduced retroperitoneal adipocyte volume by 56% (**Figure 2B**; p = 0.04). Additionally, proteomic analyses of retroperitoneal adipose tissue identified a total of 605 proteins with altered expression. After applying the inclusion criteria, 198 proteins were used in the comparison between the HFD and HFD+GbE groups. Of these 198 proteins, 20 were found to be down-regulated in the HFD+GbE group, 10 were only identified in the HFD group, and 5 were up-regulated in the HFD+GbE group (**Table 2**). Interestingly, some of the down-regulated proteins are known to participate in inflammation (mast cell protease-1, complement C3, T-kininogen 1), adipose tissue metabolism (3-ketoacyl-CoA thiolase B), and oxidative stress (peroxiredoxin-1), or related to steatosis and type 2 diabetes (fetuin-B). Additionally, cathepsin D and citrate synthase, which are associated with mitochondrial function, were found to be up-regulated in the HFD+GbE group, when compared to controls.

**Table 2:** List of proteins that were significantly altered by *Ginkgo biloba* extract treatment.

UniProt ID	Gene name	Protein description	Fold change (ginkgo/saline)	p-value
Down-regulated				
P04639	Apoa1	Apolipoprotein A-I	0.62	0.02
P01026	C3	Complement C3	0.40	0.05
Q64428	Hadha	Trifunctional enzyme subunit alpha, mitochondrial	0.58	0.04
Q9QX79	Fetub	Fetuin-B	0.53	0.04
Q5XI73	Arhgdia	Rho GDP-dissociation inhibitor 1, Rho GDI 1	0.68	0.05
P20761	Igh-1a	Ig gamma-2B chain C region	0.58	0.05
Q63716	Prdx-1	Peroxiredoxin-1, EC 1.11.1.15	0.54	0.04
Q6B345	S100a11	Protein S100-A11	0.46	0.00
Q66H98	Cavin-2	Caveolae-associated protein 2	0.40	0.04
P05545	Serpina3k	Serine protease inhibitor A3K, Serpin A3K	0.47	0.03
P00507	Got2	Aspartate aminotransferase_mitochondrial	Not detected in the HFD+GbE^1^	
P46844	Blvra	Biliverdin reductase A		
Q64573	N/A	Liver carboxylesterase 4		
Q8VHV7-3	Hnrnph1	Isoform 3 of Heterogeneous nuclear ribonucleoprotein H		
Q99PS8	Hrg	Histidine-rich glycoprotein		
P01048	Map1	T-kininogen 1		
P09650	Mcpt1	Mast cell protease 1		
P35467	S100a1	Protein S100-A1		
P07871	Acaa1b	3-Ketoacyl-CoA thiolase B_peroxisomal		
P50137	Tkt	Transketolase		
Up-regulated				
P24268	Ctsd	Cathepsin D	2.67	0.04
Q8VHF5	Cs	Citrate synthase, mitochondrial,	1.98	0.01
Q6P7Q4	Glo1	Lactoylglutathione lyase,	2.31	0.01
Q01129	Dcn	Decorin	1.65	0.04
P68370	Tuba1a	Tubulin alpha-1A chain	2.26	0.05

UniProt, Universal Protein Resource; p-value for student t test.

^1^ indicates down-regulation by GbE.

**Figure 3:**
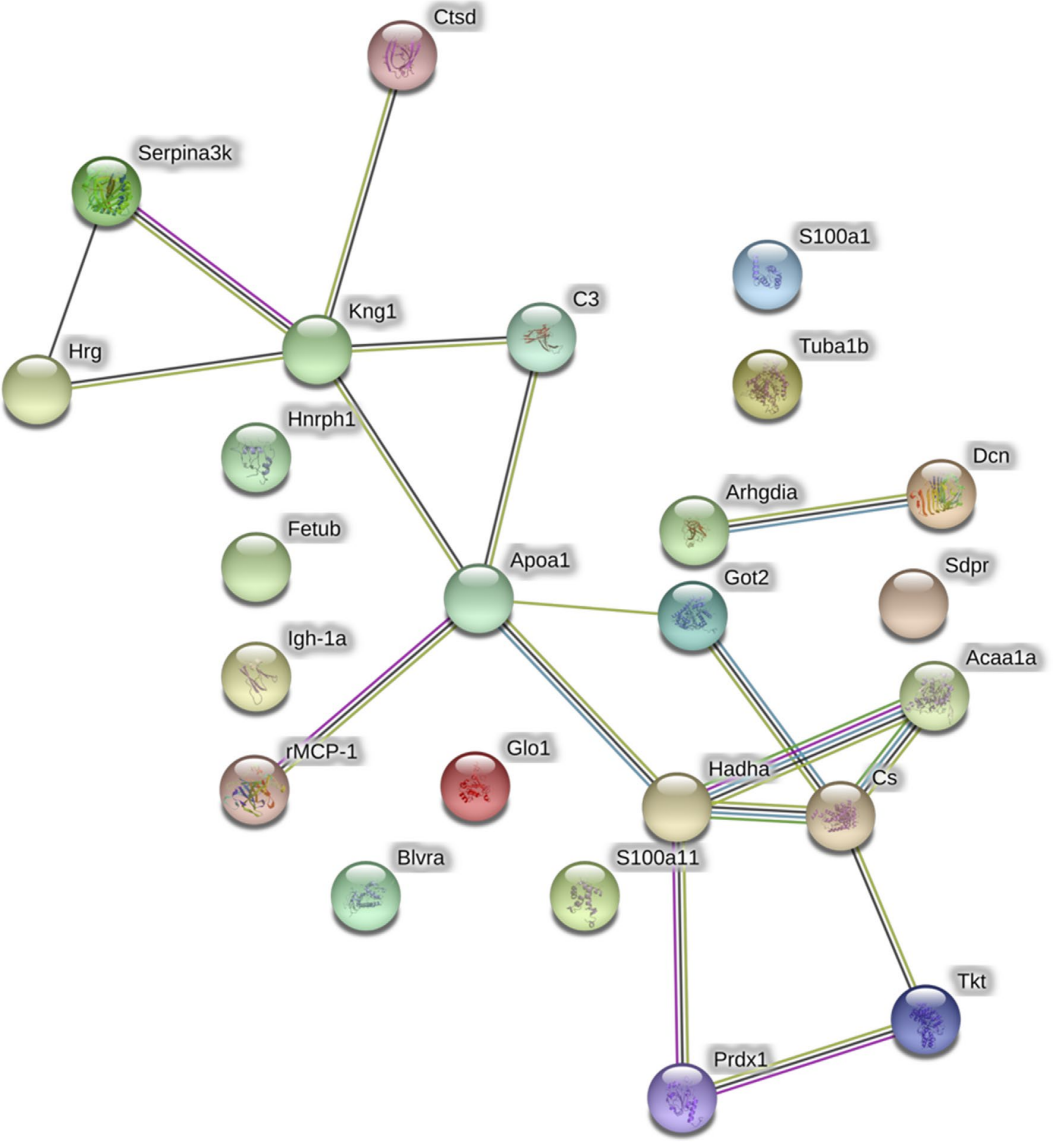
Graphic representation of the protein-protein interaction networks with significant differences between the HFD and HFD+GbE groups in retroperitoneal adipose tissue. Nodes represent individual proteins annotated with common gene names: Got2, Aspartate aminotransferase; Blvra, biliverdin reductase A; Hnrnph1, heterogeneous nuclear ribonucleoprotein H; Hrg, histidine-rich glycoprotein; Kng1, T-kininogen 1; rMCP-1, mast cell protease 1; S100a1, protein S100-A1; Acaa1a, 3-ketoacyl-CoA thiolase B; Tkt, transketolase; Apoa1, apolipoprotein A-I; C3, complement C3; Hadha, trifunctional enzyme subunit alpha; Fetub, fetuin-B; Arhgdia, Rho GDP-dissociation inhibitor 1; Igh-1a, Ig gamma-2B chain C region, Prdx-1, peroxiredoxin-1; S100a11, protein S100-A11; Sdpr, caveolae-associated protein 2; Serpina3k, serine protease inhibitor A3K; Ctsd, cathepsin D; Cs, citrate synthase, mitochondrial; Glo1, lactoylglutathione lyase, Dcn, decorin; Tuba1b, tubulin. Vectors between nodes are color coded to represent different levels of information: black, evidence of co-expression; pink, interactions experimentally determined; blue, association in curated databases; yellow, co-mentioned in PubMed abstracts; green, neighborhood in the genome.

The analysis of protein–protein interactions (**Figure 3**) depicts significant interactions among the 25 proteins affected by GbE treatment (enrichment p-value = 3.53×10^-9^). The pathway analysis indicated that GbE treatment significantly modified proteins related to oxidation-reduction process, lipid binding, fatty acid metabolism, and carbon metabolism (**Table 3**).

**Table 3:** Integrated pathway analysis of proteins that were significantly altered.

Pathway	Proteins	False discovery rate
Oxidation-reduction (redox) process	3-Ketoacyl-CoA thiolase B ↓ Apolipoprotein A-I ↓ Biliverdin reductase A ↓ Citrate synthase ↑ Trifunctional enzyme subunit alpha ↓ Peroxiredoxin-1 ↓ Apolipoprotein A-I ↓	0.0218
Lipid-binding proteins	Rho GDP-dissociation inhibitor 1 ↓ Complement C3 ↓ Aspartate aminotransferase ↓ Caveolae-associated protein 2 ↓	0.00506
Fatty acid metabolism	3-Ketoacyl-CoA thiolase B ↓ Trifunctional enzyme subunit alpha ↓ Citrate synthase ↑	0.0393
Carbon metabolism	Aspartate aminotransferase ↓ Trifunctional enzyme subunit alpha ↓ Transketolase ↓	0.00121

## Discussion

Obesity has become an emerging global epidemic, and there is a growing demand for the discovery and/or development of new therapies for this disease, as well as its related disorders. Despite the great progress in pharmaceutical research and development, ethnopharmacological approaches are gaining notoriety for their utility in the prevention and treatment of obesity. In this context, the present study aimed to further evaluate the beneficial effects of GbE on the metabolism of DIO rats. The proteomic analysis of visceral fat depots identified significant differences in the expression levels of proteins involved in lipid and carbon metabolism, inflammation, and oxidative stress, all of which have been linked to the pathogenesis of obesity-related disorders.

The non-target proteomic analyses, employed for this study, demonstrated that the GbE treatment significantly reduced the protein expression levels of peroxiredoxin, which is one of the enzymes responsible for regulating endogenous H_2_O_2_ levels. In agreement with these results, it was also observed that GbE was capable of modulating oxidative stress-related processes, especially with regards to the augmented catalase activity and reduced MDA levels in retroperitoneal fat depots. These findings are consistent with previous studies, which demonstrated that GbE or its bioactive fraction (bilobalides) displayed a protective effect against diabetic nephropathy (Lu et al., 2007), hippocampal structure damage and impaired function caused by a trimethyltin induced neurodegeneration (Kaur et al., 2013), H_2_O_2_-induced oxidative damage in human melanocytes (Lu et al., 2016), and liver fibrosis and colitis induced by carbon tetrachloride (Zhou et al., 2006). Furthermore, it has been shown that GbE affords protection against aluminum chloride-induced lipid peroxidation, and also increases the antioxidant enzyme activity in the brain and testis of male rats (Mohamed and Abd El-Moneim, 2017).

Indeed, it has been postulated that the GbE-related antioxidant and anti-inflammatory effects are due to its unique bioactive compound composition. For example, GbE flavonoids have been demonstrated to reduce inflammation by inhibiting cyclooxygenase-2, an enzyme responsible for the conversion of arachidonic acid into thromboxane and prostaglandins (DeFeudis et al., 2003; Mahadevan and Park, 2008), while bilobalides have been reported to stimulate SOD and catalase activities (Mahadevan and Park, 2008).

It should be noted that GPx and peroxiredoxin are effective at consuming subtoxic H_2_O_2_ levels, while catalase consumes toxic H_2_O_2_ levels (Rindler et al., 2013). It is possible that the observed reduction in peroxiredoxin activity occurred in response to the higher effective H_2_O_2_ consuming activity of catalase. Additionally, decreasing intracellular H_2_O_2_ concentrations ought to protect biological membranes against the lipid peroxidation, which is consistent with the demonstrated reduction in MDA levels. Furthermore, the expression of lactoylglutathione lyase (also known as glyoxalase I) was also up-regulated in the HFD+GbE group. This enzyme is a critical component of the glyoxalase system, which detoxifies methylglyoxal, a highly cytotoxic compound formed as a side-product of lipid peroxidation and glycolysis (Francisqueti et al., 2017; Frandsen and Narayanasamy, 2018). Due to the fact that the accumulation of methylglyoxal can induce chronic inflammation and oxidative stress, glyoxalase system activators might be a promising therapeutic strategy (Vulesevic et al., 2014; Allaman et al., 2015). Hence, the fact that lactoylglutathione lyase expression was increased in the HFD+GbE group, provides additional evidence for the observed antioxidant action.

In addition to evaluating the antioxidant properties of GbE, proteomic analyses revealed that GbE-treated animals displayed down-regulated expression of proteins involved in the inflammatory cascade, including: complement C3, mast cell protease-1, kininogen-1, Ig gamma-2B, and S100 proteins. In fact, there is strong evidence suggesting that obesity triggers a chronic low-grade inflammation which is linked to insulin resistance (Żelechowska et al., 2018), while complement C3 appears to be involved in adipose tissue inflammation associated with weight gain and the development of diabetes and non-alcoholic liver disease (Engström et al., 2005a; Engström et al., 2005b; Hertle et al., 2014; Xu et al., 2016). Moreover, previous studies have shown that mast cell protease levels are significantly higher in patients with obesity, resulting in impaired glucose metabolism through the inhibition of adiponectin (Wang et al., 2011; Shi and Shi, 2012; Żelechowska et al., 2018). With regards to T-kininogen-1, it is typically associated with the acute-phase inflammatory response. For example, it was demonstrated that suppression of this response with anti-inflammatory xenobiotic treatment was, at least in part, related to lower T-kininogen-1 serum levels (Joe et al., 2014).

Likewise, the GbE-induced reduction in IgG-2B chain C expression also supports an anti-inflammatory role, since this protein has been recognized as a proinflammatory protein produced by B cells (Bowden-Davies et al., 2015). Indeed, early B-cell infiltration, preceding T-cell infiltration, in the adipose tissue of DIO mice leads to macrophage accumulation and insulin resistance (Duffaut et al., 2009; Frasca et al., 2017). Remarkably, the S100 proteins were also down-regulated in the HFD+GbE group. This family of proteins has a wide range of intracellular and extracellular functions, and is considered an important regulator of macrophage inflammation, tissue damage, and oxidative stress (Xia et al., 2018). As shown in **Table 2**, some the proteins that were down-regulated in the HFD+GbE group play pivotal roles in the inflammation process, which might explain, at least in part, the reported reduction in retroperitoneal TNF-α levels, under the same experimental conditions (Hirata et al., 2015).

It is noteworthy that the proteomic analysis depicted a reduction in fetuin B expression levels following GbE treatment. Fetuin B is an adipokine/hepatokine that shares 22% homology with fetuin A, belongs to the superfamily of cysteine protease inhibitors, and shares cysteine-like domains with kininogens and histidine-rich glycoproteins (Denecke et al., 2003). Fetuin B has also been reported to be up-regulated in obese mice, and associated with the development of insulin resistance/type 2 diabetes and hepatic steatosis (Olivier et al., 2000; Miehle et al., 2018). Furthermore, using an “omics” approach with human liver samples showed that fetuin B levels were elevated in patients with liver steatosis and type 2 diabetes (Meex et al., 2015). The same study also found that silencing the fetuin B gene improved glucose tolerance in obese mice; thus, suggesting that this protein plays a role in the pathogenesis of diabetes (Meex et al., 2015). Furthermore, the reduction in fetuin B levels observed in the HFD+GbE group is consistent with previously published studies from our laboratory, which demonstrated the efficacy and benefits of GbE on insulin signaling and sensitivity (Banin et al., 2014; Hirata et al., 2015).

It was also interesting that GbE treatment down-regulated heterogeneous nuclear ribonucleoprotein H, since this protein may be involved in the production of proapoptotic regulator Bcl-xS (Garneau et al., 2005), and could account for antioxidant and anti-inflammatory properties of GbE. Moreover, serine protease inhibitor was another down-regulated protein which has been shown to be elevated in individuals with type 2 diabetes, and has been recommended as a biomarker for the early detection of the disease (Takahashi et al., 2017).

The link between transketolase and obesity has not received much attention. This enzyme has been found to be decreased in individuals with obesity, when compared to non-obese controls, and was proposed to be a compensatory mechanism for the inhibition of adipocyte enlargement (Pérez-Pérez et al., 2012). Interestingly, the results showed that transketolase is found exclusively in obese animals, which would indicate that GbE could prevent cellular expansion. Indeed, DIO animals treated with GbE displayed a 56% reduction in adipocyte volume. With regards to adipocyte volume, it is important to point out that a reduction in adipocyte volume not only promotes a reduction in adiposity *per se*, but also modulates adipokine secretion. For example, by reducing leptin and increasing adiponectin levels, cardiovascular risks are reduced and insulin signaling is improved (Lee and Shao, 2014). Smaller adipocytes are also more insulin responsive, less lipolytic and secrete fewer free fatty acids (FFAs) and inflammatory cytokines into the plasma (Yang and Smith, 2007). Since obesity-related chronic low-grade inflammation is associated with insulin resistance and type 2 diabetes (O’Connell et al., 2010), it is possible that the benefits associated with GbE on insulin sensitivity and signaling are related to the reduction in adipocyte hypertrophy and the expression of proteins involved in inflammation and oxidative stress. Indeed, we have previously demonstrated that GbE treatment significantly reduced both the epididymal adipocyte volume and *de novo* lipogenesis (Hirata et al., 2019), which allowed us to suggest that GbE might play an antiobesogenic effect.

The proteomics approach also identified proteins that were up-regulated in response to GbE treatment. For example, decorin, a small leucine-rich proteoglycan, expressed by stromal/vascular cells, has been reported to be up-regulated in tissue undergoing volume increases, since it is required for remodeling the new extracellular matrix and folding its constituents (Bolton et al., 2008; Daquinag et al., 2017). It has also been suggested that decorin has proangiogenic properties, which could be involved in adipocyte hypertrophy-induced inflammation (Bolton et al., 2008). On the other hand, studies have postulated that decorin is involved in anti-inflammatory processes, due to its binding to Complement C1q and its ability to inhibit Cq1-induced insulin resistance in the adipose tissue of obese rodents (Groeneveld et al., 2005; Zhang et al., 2007; Bolton et al., 2008). Additionally, decorin has been reported to be an adipogenesis inhibitor, and overexpressed in visceral preadipocytes (Meissburger et al., 2016). Indeed, the inhibition of visceral adipocyte differentiation has been proposed as a strategy for preventing obesity and several phytochemical treatments have successfully inhibited this phenomenon (Chan et al., 2011; Dave et al., 2012; Jang et al., 2017). In this context, GbE is a promising treatment for obesity, since it is rich in flavonoids and terpenoids (Drieu, 1986) and can effectively reduce visceral fat depot mass when administered to DIO rats (Banin et al., 2014).

Additionally, the results demonstrated a significant increase in the protein expression levels of both cathepsin D and citrate synthase in response to GbE treatment. Cathepsin D is a lysosomal enzyme that is activated during the early stages of adipocyte hypertrophy and induces proapoptotic protein activation (Masson et al., 2011; Eguchi and Feldstein, 2013; Khalkhali-Ellis and Hendrix, 2014; Houben et al., 2017). It is known that weight gain and obesity can activate cathepsin D and trigger the activation of pro-apoptotic proteins, by promoting mitochondrial dysfunction, followed by adipocyte death and oxidative stress induction. Interestingly, the catalase activity was significantly increased by GbE treatment, and these animals also presented reduced levels of MDA, providing further evidence that GbE possesses antioxidant properties. One possible mechanism to account for these observed changes is that GbE up-regulates cathepsin D expression, which will, consequently, reduce hypertrophic adipocytes *via* apoptosis and concomitantly protects against oxidative stress, due to the potent antioxidant effect of this herbal medicine. In fact, the data revealed that there was a reduction in adipocyte volume in response to GbE treatment, which is indicative of tissue remodeling. In fact, previous work demonstrated that the remodeling of adipose tissue is characterized by smaller adipocytes, secreting anti-inflammatory adipokines, thus protecting against obesity-induced inflammation and metabolic-related disorders (Choe et al., 2016).

It was also found that GbE increased citrate synthase levels in retroperitoneal fat depots of DIO rats. In the citric acid cycle, citrate synthase is a rate-limiting metabolic enzyme that is responsible for catalyzing the reaction between acetyl-CoA and oxaloacetate to form citrate (Christe et al., 2013). This enzyme is located in the mitochondrial matrix, and displays a dynamic and modulated activity (Vieira and Valentine, 2009), with a significant and inverse correlation to obesity, in terms of both activity and protein levels (Christe et al., 2013) and it is also an indicator of mitochondrial content and function. In rats fed a HFD, krill oil supplementation not only modulated carnitine palmitoyltransferase 1A (CPT I) activity, stimulating fatty acid oxidation, but also modulated citrate synthase activity as a compensatory response to maintain the entry of acetyl-CoA produced by fatty acid oxidation (Ferramosca et al., 2015). In the livers of rats fed a HFD, Wang et al. (2012) described GbE as a regulator of lipid metabolism since CPT1A gene expression was up-regulated in these animals (Wang et al., 2012). It was also previously demonstrated that GbE increases the cellular respiratory efficiency and protects mitochondrial function (Janssens et al., 1995; Janssens et al., 1999). Thus, the observed up-regulation of citrate synthase might indicate a possible effect of GbE on mitochondrial stimulation. However, since the present study did not directly evaluate this effect, other studies are necessary to better comprehend the action of GbE on mitochondrial biogenesis/function in obesity.

Aspartate aminotransferase was only detected in samples from the HFD group. This protein is coupled with PEPCK-C and participates in glyceroneogenesis, producing oxaloacetate when neither glucose nor glycerol are available (Tordjman et al., 2007). Since our previous studies demonstrated that GbE improved insulin signaling in insulin-dependent tissues of DIO rats (Banin et al., 2014; Hirata et al., 2015), it is quite plausible that improved glucose uptake might be the result of the suppressed aspartate aminotransferase synthesis observed in the HFD+GbE group.

The proteomic approach allowed us to identify in large-scale the complete set of proteins (Chandramouli and Qian, 2009) present in the retroperitoneal adipose tissue of GbE-treated obese rats and it was used as a tool for the investigation of protein-protein interactions. Regarding the 10 proteins only identified in the HFD group, we may not in principle infer that these proteins are absent in the HFD+GbE group. Instead, it is probable that the levels of these proteins in the GbE group may be below the limits of detection by the proteomics workflow employed in this work. Anyhow, the lack of detection of these 10 proteins in the GbE group is an indication of downregulation in the referred group, which remains to be clarified by future studies using more sensitive target proteomics approaches.

The present study revealed that GbE treatment was capable of modulating the expression of proteins involved in processes, such as: inflammation, lipid metabolism, and oxidative stress. Despite the limitation of the technique which do not describe how the proteins are modified, in the current study was also found that GbE stimulated catalase activity, reduced MDA levels, and reversed retroperitoneal adipocyte hypertrophy. Taken together, the findings corroborate the studies of others, and provide further evidence for the use of GbE as a therapeutic alternative for treating obesity and its related disorders since visceral adiposity, especially retroperitoneal adipose tissue, are closely linked to the development of insulin-resistance, dyslipidemia, and cardiovascular disorders. Clinical studies aimed at evaluating the efficacy and potential utility of GbE in the treatment of patients with obesity/overweight should be pursued.

## Ethics Statement

The Committee on Animal Research Ethics at the Universidade Federal de São Paulo approved all of the employed procedures for the care of the animals used in this study (Process number: 8700110814).

## Author Contributions

BH, AP and MT designed the study. BH, AP, MM, NN, BP, and RS performed the animal study and acquired the data. BH, AP, MA-V, FN, LO, ER, AT, and MT analyzed the data. BH, AP, MA-V, and MT drafted and revised the manuscript. All authors read and approved the final manuscript.

## Funding

This study was financed in part by the Coordenação de Aperfeiçoamento de Pessoal de Nível Superior–Brazil (CAPES, Finance Code 001) and by the Fundação de Amparo à Pesquisa do Estado de São Paulo (FAPESP, grant number 2014/18929-9).

## Conflict of Interest Statement

The authors declare that the research was conducted in the absence of any commercial or financial relationships that could be construed as a potential conflict of interest.
